# Review on emergency medical response against terrorist attack

**DOI:** 10.1186/2054-9369-1-9

**Published:** 2014-06-01

**Authors:** De-wen Wang, Yao Liu, Ming-min Jiang

**Affiliations:** Department of Experimental Pathology, Institute of Radiation Medicine, Academy of Military Medical Sciences, No.27 Taiping Road, Beijing, 100850 China; Director of Institute of Forensic Science, Ministry of Public Security, No.17 Muxidi Nanli, West District, Beijing, 100038 China; Director of Department of Military Medical Information, Institute of Health Logistics and Medical Information, Academy of Military Medical Sciences, No.27 Taiping Road, Beijing, 100850 China

**Keywords:** Terrorist attacks, Medical rescue

## Abstract

Terrorism is a global issue and a constant international threat. As a result, anti-terrorism and emergency response strategies are tasks of critical importance that have a direct impact on the national security of every country in the world. This paper reviews new characteristics of international anti-terrorism measures and offers an in-depth reflection on emergency medical response countermeasures; additionally, this paper presents the goals of related research, which include: 1) to present a model of a highly efficient medical response command; 2) to introduce the pre-planning phases of the emergency medical response; 3) to establish a response system capable of handling various types of terror attacks; 4) to promote anti-terrorism awareness to the general public and emphasize its prevention; and 5) to continue basic investigations into emergency medical responses for various types of terrorist attacks (for example, the classifications and characteristics of new injuries, pathophysiology, prevention and treatment of the resultant stress disorders, improved high-efficiency medical response measures and equipment, etc.).

Terrorism is an age-old plague that has only received enough attention in the international community in the wake of the “**9 · 11**” event. With the incidence of terrorist attacks and the number of casualties constantly on the rise, along with the ever-expanding harm it brings to people around the world, terrorism has become more difficult to prevent and control; thus, the fight against terrorism and emergency medical succor for victims have become important issues for all concerned countries, including China.

## Improving the understanding of the potential hazards of terrorist attacks involving nuclear, chemical and biological weapons

### Terror attacks using nuclear radiation

Terror attacks using nuclear radiation usually refer to attacks involving the use of nuclear weapons or radioactive devices. The former include “standard” nuclear bombs (atomic, hydrogen) and “coarse” nuclear bombs (“simple” nuclear weapons). Currently, nuclear materials are very easily accessible on the international market, and it is by no means challenging to make simple nuclear devices that are very powerful. Unfortunately, this has created a very favorable situation for terrorist attacks involving nuclear weapons. This category includes depleted uranium bombs (high-density, high intensity, high-hardness depleted uranium or depleted uranium alloy shells) and “dirty bombs”, which consist of radioactive substances in the form of liquid or solid particles that are spread throughout the air, water and soil through the use of conventional explosives or special simple devices detonated in a non-nuclear blast manner, resulting in severe short-term or long-term radioactive pollution. This topic deserves our vigilance because it is no longer difficult to make simple nuclear bombs, as many countries now possess nuclear weapons or have the potential to manufacture nuclear materials.

## Terror attacks using chemical weapons

Many countries around the world now possess chemical weapons. The production of high-purity chemical toxicants can be achieved using simple synthetic apparatuses. The production, possession and employment of chemical warfare agents are accessible to savage and cruel terrorists and terrorism organizations. The resulting massive casualties and mortality often lead to social turmoil and widespread panic.

## Terror using biological weapons

With the development of biological technology, it is not impossible to acquire novel, stable, pathogenic microorganisms that are highly toxic, inexpensive, and hard to detect.

In summary, current global terrorism activities have the following new traits: more diverse approaches, more unpredictable timing and locations, larger damage scales, more difficult to conduct emergency medical procedures and a farther-reaching social impact, etc. [1].

## It is extremely urgent to conduct military medical research on emergency medical procedures against terrorist attacks

It is estimated that in the next 70 years, human beings will be threatened by the following 10 hazards: terrorism, climate change, viruses, telomere loss, nuclear war, meteorite impact, robotic counter-control, cosmic rays, super-volcanoes and black holes. Of these, terrorism represents the most immediate and urgent threat. Many countries around the world, including China, are now confronting the new challenges that are brought by terrorist attacks, thus making the prevention of terrorist attacks an important and long-term task for all concerned individuals.

Providing emergency medical care during terrorist attacks is fairly different from traditional battlefield rescue, the relief of natural disasters and all sorts of accidents. Currently, several issues in emergency medical responses remain unresolved.

## Equipment for emergency medical responses need to be further modernized and serialized

The equipment for emergency medical responses should better suit different scenarios of terrorist attacks (urban area, densely populated areas and other sensitive areas). Equipment should also be more applicable to use after different sorts of terrorist attacks. At present, research and development should be focused on the following aspects: 1) emergency medical equipment for use during terrorist attacks involving nuclear, chemical and biological weapons that integrates various functions such as detection, security checks, fire control, prevention, diagnosis, treatment and evacuation; 2) monitoring and detecting instruments as well as equipment that can be used for buried victims, especially those who are deeply buried; and 3) highly efficient mining rescue equipment.

## Education of emergency medical relief personnel should be strengthened

To increase the efficiency of the emergency medical response systems, several aspects should be focused on: 1) research into the prevention of psychological problems (the prevention and intervention) caused by terrorist attacks should be further strengthened; 2) the public should be educated and trained in first aid procedures that can be used for self-rescue and mutual rescue; 3) existing achievements in military medical research should to be disseminated and made available to the public; and 4) the public’s awareness and preparedness for terrorism should be elevated.

## Emergency medical institutions and organizations need to be optimized

The establishment and optimization of emergency medical response bases should be another priority. The training and elevation of the professional skills and consciousness should also be standardized [2, 3].

## Reflections on countermeasures in emergency medical relief against terrorist attacks

### Establishment of highly efficient command for medical relief

As a non-war military action, anti-terrorism includes: 1) the prevention of terrorist attacks; 2) the minimization of consequences caused by terrorism activities; and 3) emergency medical relief [4]. Densely populated areas are likely to be chosen as targets for terrorist attacks in order to cause as much devastation and as many casualties as possible. Shortly after such a terrorist attack, there are usually massive casualties, property loss and damage, etc. As a very important part of the anti-terrorism campaign, emergency medical relief should also include the prevention of epidemics and psychological disasters in addition to rescuing the wounded. During the process of emergency medical relief, the resources of many departments should be mobilized and coordinated, including equipment, communication, transportation, epidemic prevention, medical service, civil defense, fire control and environmental protection. Thus, it is important and necessary to set up an efficient and powerful command that is capable of directing and coordinating multiple departments. The command can be put under the health authority and can be turned into a joint command that combines the forces and resources of the existing medical emergency treatment system, as well as the chemical accidents rescue organization and the military “Three Prevention” rescue organization when necessary. Meanwhile, authority should be simplified and streamlined to improve response speed. It is also essential that rehearsals should be repeatedly conducted to identify and resolve any possible problems in order to improve the coordination of the different departments.

On September 1, 2004, in the city of Beslan in the Republic of North Ossetia in northern Russia, more than 1000 students, teachers and parents were held as hostages. Thanks to the establishment of an anti-terrorism command under the direct leadership of the Russian president, a positive outcome was achieved. In response to terrorist attacks, it is very important to establish an inter-departmental rescue mechanism and conduct a coordinated command on the nation level because emergency medical relief usually involves very hard and challenging tasks that require the mobilization of all available forces and resources [5, 6].

## Perfection of various medical response contingency plans

Because terrorist attacks are usually unpredictable and sudden, without any prior signs or time to prepare, it is necessary to establish various national, provincial and municipal anti-terrorism rescue contingency plans and systems that are ready to respond to any possible terrorist attack. It is also important to establish an emergency medical defense plan at all levels, as well as monitoring and alarming systems. It is important to build up skillful professional teams that receive regular training and simulation drilling to respond swiftly to emergencies. The storage of anti-terrorism rescue protection equipment, first aid and decontamination drugs should be in proportion to the density of the population of a certain area. The public should also receive skill training for anti-terrorism medical protection to raise the public’s anti-terrorism awareness and to improve their self-rescue and mutual rescue abilities.

Additionally, special attention should be given to the design of medical relief contingency plans for terrorist attacks involving nuclear, biological and chemical weapons. The rescue organization should contain professional rescue personnel specializing in dealing with terrorist attacks involving nuclear, chemical or biological weapons. Different sizes of emergency medical response teams should be flexibly mobilized based on the type of terrorist attacks. The equipment for rescue squads and the allocation of drugs should take into account practical needs. Based on the abruptness of terrorist attacks, the aid safeguard mechanism should give equal consideration to the centralization and dispersion of rescue forces so that emergency medical relief can reach a wide coverage, with special emphasis on the extensively affected areas. Guidelines for emergency medical relief in different circumstances should be established so that first aid on the scene, wound evacuation and treatment and epidemic prevention could be carried out in a rapid, standardized and organized manner [7].

## Establishment of rescue systems responding to different types of terrorist attacks

In view of the trauma caused by terrorist attacks involving explosions, hijackings, nuclear weapons, chemical weapons and biological weapons, the search, excavation and first aid systems needed to rescue buried victims should be established as soon as possible. The emergency medical response (triage, hemostasis, bandaging, fixation, antishock, pulmonary ventilation, etc.) and delivery evacuation systems should also be established. Different rescue systems and rescue bases responding to various types of terrorist attacks should also be established. The anti-terrorism medical relief research should be conducted in a targeted manner. Anti-terrorism training should be strengthened through various classes and the dissemination of new skills and approaches to improve the anti-terrorism emergency medical response, especially to improve the ability to address s terrorist attack involving massive casualties.

In the emergency rescue after the terrorist attack, the army’s various advantages, including better discipline, sophisticated equipment, rapid responsiveness and insight into various rescue skills (especially their skills in dealing with nuclear, chemical and biological damage) should be exploited.

## Emphasis should be placed on research on the prevention of psychological problems caused by terrorist attacks

### Prevention of psychological problems caused by terrorist attacks

Terrorist activities, especially ones possibly involving nuclear, chemical and biological weapons, will likely cause severe mental strain on the public. Therefore, the prevention and research of potential psychological problems should be established as an integral part of the emergency medical response. Regular anti-terrorism drills should be held so that the general public will not panic and, thus, will better cope with a terrorist attack. In the meantime, the public’s mental power could also be gradually strengthened during any terrorist attack.

## Establishment of mental health counseling systems for emergency medical relief

The public should be taught anti-terrorism and emergency medical skills. With the help of mass media, medical institutions at different levels should publicize that psychological stress reactions to terrorist attacks are normal and instruct the public on how to carry out emergency medical self-rescue, as well as where to obtain psychological counseling service, etc. These measures could be very significant in terms of relieving the psychological effects of a terrorist attack and in preventing post-traumatic stress disorder. In view of the phobia, anxiety, depression and other mental disorders caused by terrorist attacks, mental health professionals should be promptly organized to provide mental intervention and diverse counseling services to minimize the occurrence of psychological problems.

Development of intervention countermeasures for psychological problems caused by terrorist attacks is a fundamental area of research with an urgent need. Typical approaches usually involve large-sample extensive epidemiological surveys and quantified statistics and analyses to evaluate the psychological impact of a terrorist attack on populations of different ages, genders, educational backgrounds and occupations, thus making it possible to conduct targeted research on prevention and intervention countermeasures [8, 9].

## Fundamental research into emergency medical responses to terrorist attacks

Targeted etiological studies on the injuries, diseases and new characteristics of injuries caused by terrorist attacks involving explosions, kidnapping, nuclear weapons, chemical weapons and biological weapons should be conducted and continued. For instance, new characteristics of injuries caused by new explosive devices, new methods of explosion, psychological stress of hostages, nuclide properties of dirty bombs in nuclear-related terrorist attacks, the characteristics of combined injuries caused by various factors (explosion, radiation, toxicity), the damage and the prevention of damage from new chemical warfare agents, rapid identification of biological agents and the damage and the prevention of damage from new biological agents (genetic weapon) should all be studied.

Research into the prevention of psychological stress effects caused by terrorist attacks and special multi-disciplinary research (psychology, pathology and pharmacy) should be initiated. Efforts should also be made to explore the mechanism of psychological problems caused by terrorist attacks and to work out prevention and intervention measures so that the characteristics of post-traumatic stress disorder could be determined.

In the research into new equipment that can be used for emergency medical relief against terrorist attacks, the following aspects should be addressed: 1) studies on efficient prevention measures employed at the scene of terrorist attacks involving explosions, kidnapping, nuclear weapons, chemical weapons and biological weapons should be conducted; 2) studies on new technologies, approaches and equipment that facilitate emergency rescues, such as hemostasis, bandaging, fixation, pulmonary ventilation, antishock, delivery and evacuation, at the scenes of terrorist attacks should be strengthened (special emphasis should be given to research into highly efficient life detection equipment that can be used to rescue buried victims, including multi-directional and multi-targeting life detection equipment); and 3) the research and development of non-contact monitoring equipment that can be used to detect weak life signals of the buried victims should also be accelerated.
